# Six Cases Treated Showing That Tuberculosis of the Joints Can Be Cured by Medical Treatment

**Published:** 1893-03

**Authors:** W. L. Morgan

**Affiliations:** Baltimore, Md.


### SIX CASES TREATED SHOWING THAT TUBERCULOSIS OF THE JOINTS CAN BE CURED BY MEDICAL TREATMENT.

W. L. Morgan, M. D., Baltimore, Md.

While my paper on "Indigestion" was in preparation, the April number of the *Southern Journal of Homœopathy* came to hand. On page 555, is Dr. T. E. Linn's article on "Tuberculosis of Joints—Resection of the Knee Joint. A case." In the second column of the page he says: "By surgical means we can cure the tubercular joints—by medical treatment, never." This statement is directly at variance with the claim of my paper. Then on page 560, a case is given where he resected the knee and made a permanent cripple of the patient. Yet the operation was a success he says—a stiff leg. Thus it makes it necessary for me, in defense of my position, to report a few cases of the same kind treated and cured by strictly medical treatment, one of which the finest surgical treatment in America failed to cure, which, under medical treatment, is now rapidly approaching good health with fair use of the joint. Three others were perfectly cured.

My first case was treated in 1873. It was Miss L. H., of Marion County, Indiana, age twenty years. Two years before in jumping from a carriage she struck her knee against a stone. Soon after enlargement commenced. It was now more than double the size of the other knee, with very slight movement of the joint. Her knee had been examined by a number of eminent surgeons, all of them agreeing that it was a case of exostosis. One recommended an operation by cutting away the bone; others different kinds of surgical treatment. They gave her a printed picture of exostosis of both tibia and femur, showing large growths of bone, which they said should be cut off by surgical operation. All agreed that it was tubercular.

A prescription, Cal.-phos.$^{30}$ being the principal remedy, was given her. Met her six months later walking with a cane; three months after that time she was entirely well. She married; three years afterward she and her two children were in excellent health.

Baltimore, 1882.—Met Mrs. B. from Middlesex County, Virginia, with her son, Willie, suffering with hip disease. She had been to two hospitals for advice. Got no encouragement for a cure, though they advised a hip-joint operation, but gave no hope of his recovery. Willie was six years old, light hair, blue eyes, fair skin, body emaciated, two scrofulous ulcers on his neck, thigh flexed at right angles with the body, heel solid against nates, knee and hip joints stiff, large ulcer near the great trochanter and three others outside of the thigh. Head large, eyes bright, abdomen very large, canine appetite, stools undigested, smelling badly, food passed thirty minutes after eating, the pit of the stomach convex instead of concave. He got Calccarb.$^{200}$, a drachm vial, six number twenty pellets once a day. Gave no other medicine. Completely cured in one year. Now Willie is a live, active young man, managing a truck farm with no inconvenience, except a high heeled shoe.

A similar case to the one mentioned above was a girl treated by letter and cured in six months, same county.

By letter a similar case to the above, in the same neighborhood, was a colored boy now under treatment and reported improving rapidly.

1890. Mrs. H., age thirty-five, married, no children. For twenty years has been suffering from caries of the right innominata bone below the acetabulum, with large ulcers, external and internal, passing through the rectum. Right limb about four inches shorter than the left; walks with a high-heeled shoe. Formerly suffered great pain, most of the time unable to attend to household duties. Suffered from night-sweats mostly about the neck and chest. Sweaty feet, raw between toes. Gave Sil.$^{m}$ and continued. Since then have used Calc-carb., but now on Asaf.$^{30}$. Has been for a year able to attend to all household work. Walks on streets, gradually approaching good health. Has made wonderful improvement for a person of her age with a disease of such long standing.

Willie E., age six years, light hair, blue eyes, large head, very large abdomen, limbs wasted, flabby skin, and convex stomach; caries of the left innominata at the upper end of the sacro-iliac-symphysis with a large ulcer. This patient had been an inmate of one of the finest hospitals in America for ten months. Had had three surgical operations in that time, and was preparing for another when his father took him away, after which time I was called to attend him. Found him as above described, with a large cicatrix perpendicularly through the nates, three inches long and three-fourths wide, with a fistula opening near the upper end with a piece of oiled silk folded two inches deep into it, with extensive bandaging, with antiseptics in great abundance.

My first work was to remove all this and apply absorbent cotton and sufficient bandage to hold it and absorb the pus which was flowing in great quantity, thin and varnish color. He got Calc-carb.$^{m}$. Continued for thirty days. I directed him to go home to his mother in Southwest Virginia. Six weeks later received a letter that Willie was doing finely, improved in every way, gained nine pounds in weight. Continued. Reports continue favorable and continued improvement.

In all these cases the bone affection had been long preceded by some derangement of the digestive organs, which continued through the case and furnished the leading indications for the remedy in each case, and all were benefited and half cured without the loss of the joints.

I suppose many of the members of this Association can report numerous cases similarly treated with like results, and can join me in the opinion that tuberculosis of the joints may be cured by medical treatment, but *by surgery, never.*

Also, I protest against subjecting the sufferers already afflicted to the worse suffering caused by the loss of joint, limb, or life under the idea of being cured by a surgical operation.

The following cases were reported to me by Dr. J. C. Hammer. One I knew myself.

Mr. H. S., age fifteen. Had been treated ten years for hip disease, and given up as hopeless.

Cured by a homœopathist in three years. Now, eight years later, good health.

Miss Martha L. had hip disease five years—a bad cripple. Cured by a homœopathist in one year.

Mrs. J. C., Buckinghan County, Va. Hip disease from childhood. Treated by letter and cured in two years by a homœopathist.

Miss Gracie B., Loudon County, Va. Hip disease from infancy. Cured in eighteen months by homœopathic treatment.

All the above cases were preceded by disease of the digestive organs.

